# On Placenta Prævia

**Published:** 1871-02

**Authors:** T. Gaillard Thomas


### On Placenta Prævia:

By T. Gaillard Thomas, M. D.

*American Journal of Obstetrics and Diseases of women and children.*

Dr. Thomas relates eight cases of this complication, and urges the rapid induction of labor.

"The dangers attendant upon the condition develop themselves most markedly in the first stage of labor, and death not unfrequently occurs before the os externum is dilated to a size not greater than a Spanish dollar. At this time the surgical interference, if resorted to to accomplish delivery, often destroys the lives which it is intended to save. The hand thrust too soon through a rigid os will often rupture its walls, while a delay, without the adoption of the means capable of controlling hemorrhage will necessarily favor the occurrence of a fatal result.

"On the other hand, should full dilation of the os have taken place, and the patient be exhausted from sanguineous loss, the practice of artificial delivery will not rarely be followed by a fatal prostration.

"There is no question in my mind of the fact that when it becomes the recognized practice to resort to *premature delivery*, as a prophylactic measure in these cases, the statistics which have been quoted will be very much improved upon. By resorting to this measure, we should be dealing with a woman who is not exhausted by repeated hemorrhages; the obstetrician would be in attendance at the commencement of the labor, and he would be able by hydrostatic pressure to control flooding, while the same pressure accomplished rapidly and certainly the first stage of the labor.—*Half Yearly Abstract.*

[The induction of labor before full term, making the labor entirely an artificial one, in cases where the placenta prævia is to be expected, is the doctrine advocated and taught also by Dr. James P. White, of this city.—Ed.]

